# Prospective Evaluation of a Circulating Tumor Cell Sensitivity Profile to Predict Response to Cisplatin Chemotherapy in Metastatic Breast Cancer Patients

**DOI:** 10.3389/fonc.2021.697572

**Published:** 2021-06-25

**Authors:** I. E. de Kruijff, A. M. Sieuwerts, N. Beije, W. J. C. Prager - van der Smissen, L. Angus, C. M. Beaufort, M. N. Van, E. Oomen - de Hoop, A. Jager, P. Hamberg, F. E. de Jongh, J. Kraan, J. W. M. Martens, S. Sleijfer

**Affiliations:** ^1^ Department of Medical Oncology & Cancer Genomics Netherlands, Erasmus MC Cancer Institute, Erasmus University Medical Center, Rotterdam, Netherlands; ^2^ Department of Medical Oncology, Franciscus Gasthuis & Vlietland, Rotterdam, Netherlands; ^3^ Department of Medical Oncology, Ikazia Ziekenhuis, Rotterdam, Netherlands

**Keywords:** metastatic breast cancer, circulating tumor cells (CTCs), mRNA profile, cisplatin, cDDP, resistance

## Abstract

**Background:**

Cisplatin (cDDP) has regained interest for metastatic breast cancer (MBC) patients, given the platinum sensitivity in subtypes and better manageable toxicity. Here, the primary aim was to determine whether molecular characteristics of circulating tumor cells (CTCs) could identify patients responding to cDDP and to describe the outcomes to cDDP monotherapy in a large group of MBC patients pretreated with anthracycline- and taxane-based treatments.

**Methods:**

Based on cell line data, a CTC-cDDP-sensitivity profile was generated. Applying an A’Herns single-stage phase II design, further investigation was considered worthwhile if 5/10 patients with a favorable profile responded to cDDP. Patients received 70mg/m^2^ cDDP every three weeks, CTCs were enumerated and the CTC-cDDP-sensitivity profile was determined. In total, 65 heavily pretreated MBC patients (77% received ≥2 lines of previous chemotherapy for MBC) were eligible for the per-protocol analysis. Primary endpoint was response rate, secondary endpoints included best observed response, progression-free survival (PFS) and overall survival (OS).

**Results:**

The best observed response during cDDP therapy was a partial response in 7% and stable disease in 56% of the patients. None of the patients with a favorable CTC-cDDP-sensitivity profile had a response. The median baseline CTC count was 8 (range 0-3254). Patients with <5 CTCs had a better PFS and OS than patients with ≥5 CTCs (median PFS 4.5 months (95%CI 2.38-6.62) *vs*. 2.1 months [(95%CI 1.34-2.80)(*p*=0.009)] and median OS 13.1 months (95%CI 9.89-16.33) *vs*. 5.6 months [(95%CI 3.60-7.64)(*p*=0.003)]. No other factors than CTC count were associated with outcome to cDDP therapy, including triple-negative breast cancer versus ER-positive tumors.

**Conclusions:**

The CTC-cDDP-sensitivity profile was unable to select patients responding to cDDP monotherapy. In an unselected group of heavily pretreated MBC patients, cDDP yields outcomes comparable to other chemotherapeutic regimens for heavily pretreated MBC patients. CTC count was the only factor associated with outcome in these patients.

**Clinical Trial Registration:**

(https://www.trialregister.nl/trial/3885, identifier NTR4046)

## Background

For patients with metastatic breast cancer (MBC), several systemic therapies are available, aiming to prolong survival with an acceptable quality of life. Despite the fact that only for eribulin evidence exists for superiority over other regimens from randomized trials (1, 2), multiple agents are used in anthracycline- and taxane-pretreated patients.

Agents that are increasingly used are platinum derivatives. One of these derivatives is cisplatin [*cis*-diamminedichloroplatinum (II), cDDP], an alkylating agent clinically available since the 1970s that is still being used in a wide range of tumor types. Most studies evaluating the effect of cDDP monotherapy in MBC are from the 1980s. Small phase-II studies reported response rates (RR) of 47-54% in previously untreated patients (3, 4) and of 15-21% in heavily pretreated patients (5, 6). Although the outcomes for cDDP in the first line are comparable with other chemotherapeutic agents applied in MBC, its side-effects prevented implementation into the clinical practice. However, the use of cDDP regained interest since its main toxicities, in particular nausea/vomiting and nephrotoxicity, can be handled much better nowadays. Also, there is improved insight into the tumor biology, which suggests subtypes of patients exist with tumors displaying a high sensitivity to platinum-based therapies (7–9).

Therefore, a method to select patients who will benefit from cDDP therapy is highly needed. Molecular characteristics of tumor cells can be associated with outcome to certain agents. Most molecular characterization is performed on primary tumor material. However, since the characteristics of the primary breast tumor and metastatic lesions can change over time and under treatment pressure (10), metastatic tumor cells should be explored for characteristics predicting outcome. However, obtaining tissue from metastatic lesions is an invasive and often painful procedure and sometimes impossible because of inaccessible lesions. Circulating tumor cells (CTCs), which can be repeatedly isolated from peripheral blood, represent an attractive alternative. Besides CTC enumeration, which is a proven prognostic marker in MBC (11–13), characterization of these CTCs is also possible (14–17). The characteristics of CTCs resemble the characteristics of the metastatic lesions better than that of the primary tumor (18). Therefore, characterization of these CTCs can be a promising tool to select patients who are sensitive to cDDP therapy.

The primary aims of this study were to determine whether a CTC gene expression profile based on cell lines enabled the identification of patients responding to cDDP and to describe the outcomes to cDDP monotherapy in a large group of MBC patients pretreated with anthracycline- and taxane-based treatments. 

## Methods

### Cell Line Data

Breast cancer cell line cells (regularly tested for Mycoplasma) were cultured in their respective growth media until near confluence before being plated in a 96-wells plate or added to 7.5mL blood of a healthy donor. The identity of all 17 cell lines used in this study were routinely validated by short tandem repeat (STR) analyses (PowerPlex 16 system, Promega, Madison, WI, USA). For determining the IC50 cisplatin sensitivity, cells were plated at a density of 1,000 to 10,000 cells per well in complete growth medium in the absence or presence of increasing concentrations of cisplatin (3x10-11 to 1x10-5 M). Cisplatin was dissolved in phosphatate-buffered saline and four days later cells were analyzed with the Sulforhodamine B (SRB) assay to quantify the percentage of cells remaining. IC50-values were calculated based on these data. Cell lines were classified based on their IC50 as cDDP sensitive (+2 standard deviation (SD) from the median) or resistant (-2 SD). Based on this classification, there were five cDDP resistant cell lines (T47D, SUM185, MM-453, CAMA-1 and BT-474) and eight sensitive cell lines (MM-468, SUM149, SUM52, SUM229, BT20, HCC-1937, UACC893 and SKBR-3, see **Supplementary Figure 1**). To evaluate the mRNA expression profiles, 50 cells of each cell line were spiked into 7.5mL EDTA blood of a healthy donor and enriched by CellSearch as described below. For both the cisplatin IC50 determination and the generation of the cisplatin sensitivity profiles, cell lines were analyzed in at least two independent experiments.

### CTC-cDDP-Sensitivity Profile on Cell Lines

To identify a CTC mRNA profile associated with outcome to cDDP, the gene expression data of our previously described panel of 93 genes (17) (as described below) were analyzed in the eight sensitive versus five resistant cell lines with the Diagonal Linear Discrimination Analysis (DLDA) Class Prediction tool (v4.4.1) of Biometric Research Branch ArrayTools (BRB-ArrayTools, http://linus.nci.nih.gov/BRB-ArrayTools.html) using p<0.05. The DLDA-predictor model in combination with a leave-one-out cross-validation method to compute the miss-classification rate was applied to identify a set of genes significantly differently expressed between the sensitive and resistant breast cancer cell lines to generate the CTC-sensitivity profile.

### Patient Data

**Figure 1:**
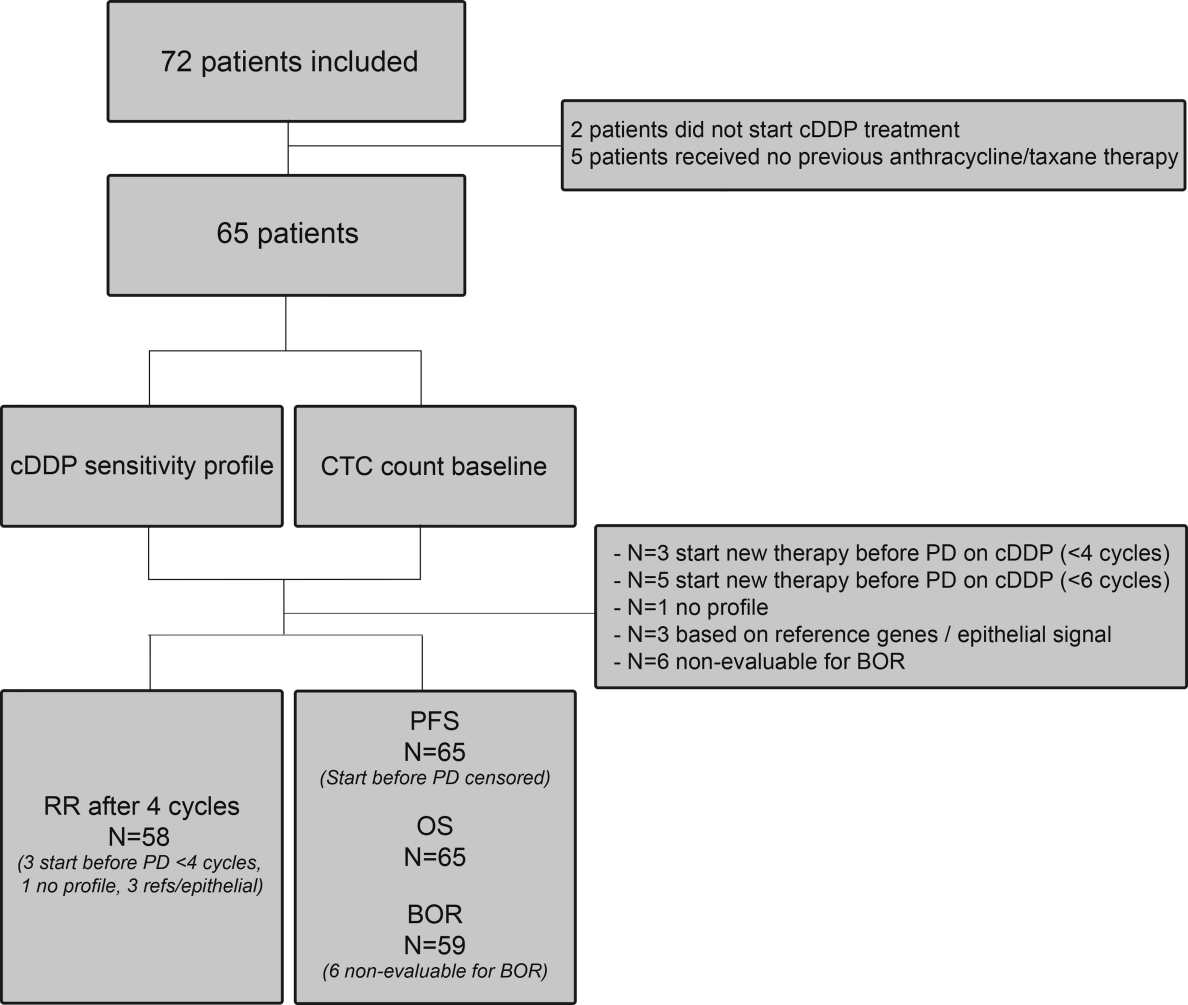
Flow chart. Flow chart of all patients included in the study. RR, response rate; PFS, progression-free survival; BOR, best observed response; OS, overall response; PD, progressive disease; cDDP, cisplatin.

The CTC-cDDP study (Dutch Trial Register NTR4046) was a prospective international multicenter trial in the Netherlands and Belgium. In this study, 72 MBC patients who had at least been pre-treated with anthracycline- and taxane-based chemotherapy and were deemed fit enough for cDDP therapy by their treating physicians were included. For the complete in- and exclusion criteria see **Supplementary Table 1**. A flowchart of the included patients is shown in **Figure 1**. The dose of cDDP therapy was 70 mg/m^2^ every three weeks and treatment continued until progression of disease, unacceptable toxicity or if patients wished to stop, with a maximum of six cycles. Treatment delay up to two weeks and dose reductions were permitted. Blood was drawn for CTC enumeration and characterization before start of cDDP therapy. Toxicity was recorded according to the National Cancer Institute Common Toxicity Criteria (CTCAE) version 4.0. Computed Tomography (CT)-scans were performed at baseline and after the second, fourth and sixth cycle and were assessed according to the Response Evaluation Criteria in Solid Tumors (RECIST) version 1.1 (19). Treatment responses according to RECIST were assessed by the radiologist of the hospital and verified by one of the authors (I.K./N.B). The study was approved by the Medical Research Ethics Committee of the Erasmus MC and local Institutional Review Boards (METC 13-007). All patients provided written informed consent.

### CTC Enumeration and mRNA Isolation

Two tubes of blood were collected from all patients before start of cDDP treatment: 7.5mL of CellSave blood for CTC enumeration and 7.5mL EDTA blood for CTC characterization. Both tubes were processed with the CellSearch system (CellSearch enumeration kit and CellSearch profile kit; Menarini-Silicon Biosystems, Huntington Valley, PA, USA). CellSave blood was processed within 96 hours and EDTA blood within 24 hours. For CTC characterization, a detailed description has been published previously (17, 20). In short, mRNA was isolated with the AllPrep DNA/RNA Micro Kit (Qiagen, Germantown, MD, USA). Thereafter, cDNA was generated and pre-amplified for the targets of interest, and real time amplified by quantitative Reverse Transcription Polymerase Chain Reaction (RT-qPCR) using Taqman Gene Expression Assays (Applied Biosystems, Carlsbad, CA, USA).

### Sample Processing and Normalization

To establish the quality of the mRNA samples of the 17 cell lines spiked into blood as well as the 70 patient mRNA samples, three reference genes (*GUSB*, *HMBS* and *HPRT1*) were added to the previously described 93-gene breast cancer profile (17). If the average reference signal of a sample was ΔC*_q_* >26.5, it was considered to be of insufficient cDNA quantity and/or quality and therefore excluded (*n*=2). Furthermore, to ensure that the expression of the genes was CTC-specific, the 12-gene epithelial profile that was established before (17), was applied to these samples. This epithelial profile has been selected from CTC samples of 910 breast cancer samples and 20 samples from healthy blood donors (HBD) to guide the selection of samples with adequate, CTC-driven RNA signal. A cut-off of -131 ΔC*_q_* (sum of the 12 genes) was applied to select samples with at least one CTC. Samples with an epithelial cut-off below -131 were therefore excluded (*n*=1).

Of the 93 genes, 55 genes are known to have a higher expression in the CTC samples than in the contaminating leukocyte background that is present after isolation of CTCs with the CellSearch system (17). In the cDDP-treated patients with sufficient cDNA quantity and quality, the 93 genes were measured, and the CTC-sensitivity profile determined, as was generated based on the cell line data.

### Statistical Analysis

The sample size for this study was based on the response to cisplatin in CTC-cDDP sensitive patients. Since RRs of 15-21% have been reported in unselected, heavily pretreated patients, a RR of 20% in the CTC-cDDP sensitive patients was deemed too low to justify further exploration (p0) in a phase III trial. A RR of approximately 60% in MBC patients with ≥5 CTCs and a favorable cDDP-sensitivity profile was considered high enough to justify further testing (p1). Applying an A’Herns single-stage phase II design to the cohort of patients with ≥5 CTCs, sufficient reference signal (ΔC*_q_* <26.5), an epithelial profile >-131, and a favorable cDDP-sensitivity profile, with p0 = 20%; p1 = 60%, α = 0.05 and β = 0.20, implied that ≥5 out of 10 evaluable patients should achieve a response to warrant further testing. Therefore, inclusion continued until 10 evaluable patients with ≥5 CTCs and a favorable cDDP-sensitivity profile were included.

The primary endpoint of this study was the RR per RECIST of patients with a favorable CTC-cDDP profile after four cycles of cDDP. All patients who had received at least one cycle of cDDP treatment were considered for the primary objective. Patients with progressive disease (PD) at the evaluation following two cycles of cDDP were considered having PD at the primary endpoint. Patients who went off study due to toxicity before the assessment following four cycles were considered ineligible for the primary endpoint and patients who went off study prior to this assessment for reasons other than toxicity were considered as having PD. The only exception were patients who switched therapy without objectified PD on cDDP therapy. These were excluded for the primary endpoint if the new therapy was started before the fourth cycle and censored at the moment of start of the new therapy for the secondary endpoints.

Secondary endpoints included progression-free survival (PFS) and overall survival (OS). PFS was defined as the time between start of treatment and progression of disease. OS was defined as time between the start of treatment till death of any cause. We also objectified the best observed response on cDDP therapy for all patients as secondary endpoint, which is the best response during therapy recorded from the start of the study treatment until disease progression or stop of treatment (according to RECIST). This was determined as complete response (CR), partial response (PR; confirmed or unconfirmed if this was the last response measurement), stable disease (SD) longer than six weeks or progressive disease (PD). All analyses were carried out in the per-protocol population.

Survival analysis were studied with the log-rank test and visualized with Kaplan Meier plots. Furthermore, univariate and multivariate Cox proportional hazards analyses were performed. For multivariate analyses, only the significant variables (P<0.05) from univariate analyses were added to the model. All computations were performed using R (version 3.4.1) and all reported p-values are two-sided.

## Results

### Preclinical Cell Line Model

To evaluate the gene expression profiles of cell line cells with a known cisplatin sensitivity, 50 cells per cell line were spiked into EDTA blood of a healthy blood donor prior to CellSearch enrichment, RNA isolation and RT-qPCR analysis. The DLDA test resulted in the following formula to identify resistant cells based on the expression levels of 9 genes: -1.3201*KRT7-0.4157*KRT17+0.5381*ERBB3-0.488*PTRF+0.4452*TFF1+0.4281*TFF3-0.4613*EGFR+0.37*TNRC9-1.0933*IGFBP3. Using optimal binning, a threshold of 7.9 was calculated to identify cisplatin resistant cells. Results were validated in an independent spike-in experiment encompassing the same cell line cells. The sensitivities and specificities for the discovery and validation experiments are given in **Supplementary Table 2** and the distribution of the cell line cells after applying our 7.9 cut-off in **Supplementary Figure 2**. To ensure that the created CTC-sensitivity profile could also be detected in patient samples, we retrospectively looked into our CTC mRNA profiling data from previously published studies (17, 21, 22). Based on these data (*n*=432), the profile was detected in around 35% of the patients with ≥5 CTCs present.

### Patient and Cycle Characteristics

In total, 72 patients signed informed consent for this study. Two patients did not start cDDP therapy due to rapid deteriorating clinical condition; five patients did not previously receive anthracycline and/or taxanes therapy. Consequently, per-protocol analysis was performed on 65 patients. Of these, 72% had ER (estrogen receptor)-positive breast cancer, the others had triple-negative breast cancer (TNBC). Most patients (77%) had already received ≥2 lines of chemotherapy for metastatic disease. Full patient characteristics are presented in **Table 1**. The median number of cDDP cycles these patients received was three (range 1-6). In total, 14 patients (22%) completed all six cycles of cDDP. There were nine patients who stopped treatment due to toxicity (six with objectified toxicity) and six patients who wanted to stop treatment in general.

**Table 1:** Patient characteristics (*n*=65).

** **	N	%	** **	N	%
**Age**			**BRCA mutation**		
≤40	6	9.2	Positive	8	12.3
41-55	24	36.9	Negative	16	24.6
>55	35	53.9	Unknown	41	63.1
**WHO performance status**			**Previous (neo)adjuvant chemotherapy**		
0	16	24.6	Yes	45	69.2
1	46	70.8	None	20	30.8
2	3	4.6			
			**Previous adjuvant endocrine therapy**		
**Menopausal status**			Yes	30	46.2
Premenopausal	10	15.4	None	35	53.8
Perimenopausal	9	13.9			
Postmenopausal	45	69.2	**Number of previous palliative chemotherapy agents**		
Unknown	1	1.5	0	5	7.7
			1	10	15.4
**BR grade**			2	18	27.7
1	0	0.0	3	17	26.2
2	16	24.6	4	10	15.4
3	27	41.5	5	3	4.6
Unknown	22	33.9	6	2	3.1
**ER status**			**Number of previous palliative endocrine agents**		
Positive	47	72.3	0	27	41.5
Negative	18	27.7	1	11	16.9
			2	14	21.5
**PR status**			3	5	7.7
Positive	33	50.8	4	5	7.7
Negative	32	49.2	5	2	3.1
			6	1	1.5
**HER2 status**					
Positive	2	3.1	**PARP-inhibitor received previously**		
Negative	62	95.4	Yes	5	7.7
Unknown	1	1.5	None	60	92.3
**Subtype**					
ER+/HER2-	44	67.7			
ER+/HER2+	2	3.1			
Triple negative	18	27.7			
Unknown	1	1.5			

Patient characteristics for all 65 patients. ER, estrogen receptor; PR, progesterone receptor; HER2, human epidermal growth factor 2.

### Response to cDDP in Patients With Favorable cDDP-Sensitivity Profile

The primary aim of this study was to determine if the CTC-sensitivity profile we determined in cell lines could predict the RR after four cycles of cDDP therapy. Seven patients could not be evaluated for the primary objective: in four patients the sensitivity profile could not be determined [lack of mRNA quality (*n*=3) or EDTA blood had not arrived <24 hours (*n*=1)], and three patients received a new therapy before they had progression on cDDP therapy. Of the 58 eligible patients, ten patients had ≥5 CTCs and a favorable CTC-sensitivity profile. None of these patients had a response after four cycles of cDDP therapy. Median PFS in these patients was 2.0 months (95%CI 0.47-3.47) and median OS 3.1 months (95%CI 0.66-5.52). The best observed response was SD in 50% (5/10) of the patients.

Median PFS in all 58 patients was 2.5 months (95%CI 1.84-3.16) and median OS 6.9 months (95%CI 3.80-9.94). The CTC-sensitivity profile in relation to PFS and OS is shown in **Supplementary Figure 3**.

### Outcomes in the Full Cohort

Six patients were non-evaluable for the best observed response, as they had to stop cDDP treatment due to toxicity, leaving 59 patients. The best observed response was a PR in 7%, while 56% had SD and 32% experienced PD (5% not evaluable). Median PFS and OS for all cDDP-treated 65 patients was 2.5 months (95%CI 2.21-2.79) and 6.9 months (95%CI 4.08-9.78), respectively.

**Figure 2:**
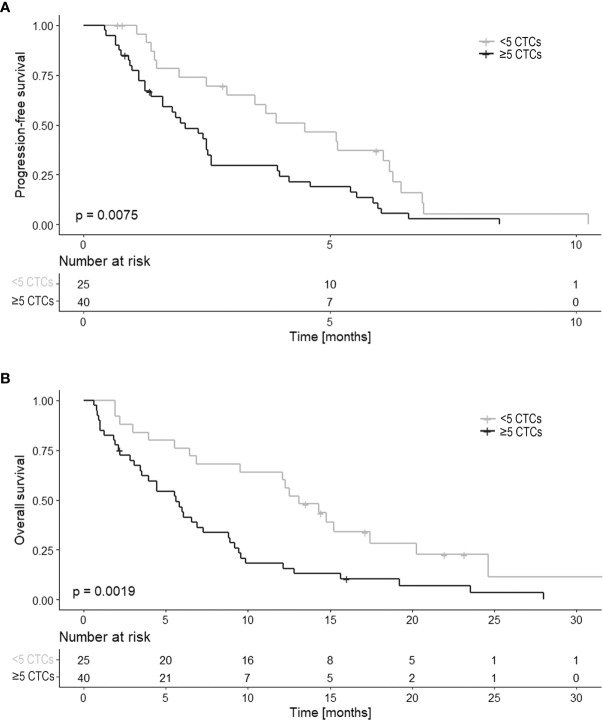
PFS and OS in relation to the CTC count (n=65). Kaplan Meier curves of **(A)** progression-free survival (PFS) and **(B)** overall survival (OS) in relation to CTC count at baseline. CTC counts are divided into two categories of < 5 CTCs and ≥ 5 CTCs.

The median number of CTCs at baseline was 8 (range 0-3254) in all 65 patients. The patients were divided into two groups: <5 CTCs (*n*=25) and ≥5 CTCs (*n*=40). Comparing these two groups showed that patients with <5 CTCs had a significantly longer PFS and OS than patients with ≥5 CTCs (HR 2.10, 95%CI 1.21-3.65, *p*=0.009 and HR 2.38, 95%CI 1.36-4.18, *p*=0.003 respectively; **Figure 2**).

### Evaluation of Other Prognostic Factors

Established prognostic factors (CTC count (<5 CTCs/≥5 CTCs), subtype, BRCA-status, BR (Bloom-Richardson) grade, previous lines of palliative chemotherapy, previous lines of palliative endocrine therapy, presence of visceral metastasis, WHO status and age) were compared in relation to PFS and OS. Only a CTC count of ≥5 was associated with a shorter PFS in univariate analysis (HR 2.10, 95%CI 1.21-3.65, *p*=0.009, see **Table 2A**). Therefore, no multivariate regression analysis could be performed. For OS, in univariate analysis CTC count and the previous lines of palliative chemotherapies were associated with outcome. When adding these variables to the multivariate analysis, both were independent prognostic factors for OS (CTC count ≥5 (HR 2.22, 95%CI 1.26-3.90, *p*=0.006) and higher number of palliative chemotherapies [HR 1.96, 95%CI 1.12-3.44, *p*=0.019)] (see **Table 2B**).

**Table 2:** Univariate and multivariate Cox Regression analysis.

(A)						
** **	Univariate analysis			Multivariate analysis		
Variable	HR	95% CI	p-value	HR	95%CI	p-value
**CTC count**	**2.098**	**1.21-3.65**	**0.009**	**2.098**	**1.21-3.65**	**0.009**
**Subtype**	0.765	0.42-1.38	0.373			
**BRCA**	0.464	0.18-1.22	0.119			
**BR grade**	0.677	0.35-1.32	0.251			
**Palliative chemo**	0.949	0.57-1.60	0.844			
**Palliative endo**	0.820	0.49-1.39	0.458			
**Visceral metastases**	0.846	0.36-1.99	0.700			
**WHO**	0.998	0.56-1.78	0.995			
**Age**	1.022	0.99-1.05	0.145			
**(B)**						
** **	**Univariate analysis**			**Multivariate analysis**		
**Variable**	**HR**	**95% CI**	**p-value**	**HR**	**95%CI**	**p-value**
**CTC count**	**2.381**	**1.36-4.18**	**0.003**	**2.219**	**1.26-3.90**	**0.006**
**Subtype**	0.974	0.54-1.74	0.928			
**BRCA**	0.512	0.18-1.43	0.200			
**BR grade**	0.568	0.29-1.11	0.100			
**Palliative chemo**	**2.139**	**1.22-3.75**	**0.008**	**1.958**	**1.12-3.44**	**0.019**
**Palliative endo**	0.800	0.47-1.36	0.411			
**Visceral metastases**	1.522	0.65-3.59	0.338			
**WHO**	0.955	0.51-1.79	0.885			
**Age**	1.026	1.00-1.06	0.060			

Univariate and multivariate Cox regression analysis (n=65). **(A)** shows all variables in relation to PFS and **(B)** in relation to OS. CTC count was analyzed as dichotomized variable (<5 CTCs/≥5 CTCs) and age as continuous variable. For subtype patients were divided in ER+ versus TNBC and for BR (Bloom-Richardson) grade all patients were grade 2 or 3. Palliative chemotherapy was divided in 0-2 and 3-6 lines of chemotherapy for advanced breast cancer. Palliative endocrine therapies were divided in 0-1 and 2-6 lines of endocrine therapies for advanced breast cancer. WHO stands for WHO performance status and was divided in WHO 0 or WHO 1-2. HR, hazard ratio. The significant values are shown in bold.

As shown in the univariate analysis, there was no difference in PFS (*p*=0.373) nor OS (*p*=0.928) between the patients with TNBC and ER+ primary breast cancer in relation to cDDP therapy. Median PFS in the ER+ patients (*n*=47) was 2.5 months (95%CI 1.83-3.17) and median OS 7.3 months (95%CI 3.68-10.90). TNBC patients (*n*=18) had a median PFS of 2.9 months (95%CI 1.66-4.18) and OS of 6.1 months (95%CI 5.12-7.04) (**Supplementary Figure 4**). The BRCA status was known in 24 patients. Between BRCA-positive (*n*=8) and BRCA-negative (*n*=16) patients, no difference was found in PFS (*p*=0.119) and OS (*p*=0.200). Median PFS in the 8 patients with a known BRCA-mutation was 4.5 months (95%CI 0.00-10.47) and median OS was 9.6 months (95%CI 0.00-20.98). For the 16 patients without BRCA-mutation, median PFS was 2.6 months (95%CI 2.46-2.74) and median OS 6.6 months (95%CI 5.26-7.94).

### Toxicity of cDDP Therapy

All serious adverse events (SAEs) and all adverse events (AEs) of grade ≥3 were reported in all patients who received ≥1 cycle of cDDP (*n*=65). In total, 119 SAEs were reported; in 27 patients, no SAEs were reported. The following SAEs were reported five times or more: nausea, dyspnea, acute kidney failure, anemia, and hypercalcemia. A line listing of all SAEs is shown in **Supplementary Table 3**. Of the 119 reported SAEs, only 12 of the SAEs (10%) were grade 3 or higher and related to cDDP treatment.

## Discussion

The data presented here shows that the CTC-cDDP-sensitivity profile was unable to select patients who will respond to cDDP treatment. The primary aim with a RR of approximately 60% in MBC patients with ≥5 CTCs and a favorable cDDP-sensitivity profile, was set relatively high given the relatively expensive and complex handlings to enumerate and characterize CTCs. However, none of the patients with a favorable profile had a response to cDDP therapy. The CTC-cDDP-sensitivity profile was generated based on 17 breast cancer cell lines which were thought to represent the clinical breast cancer subtypes. However, it could be that these cell lines were not representative enough. Also, since breast cancer is a heterogeneous disease, it can be difficult to generate a profile that predicts response for all breast cancer subtypes. Furthermore, only 55 genes in the measured CTC mRNA profile were CTC-specific. This selection of genes might have been too limited for accurate prediction of cDDP sensitivity, or relevant genes related to cDDP sensitivity might have been excluded from the mRNA profile because their expression in CTCs does not significantly exceed their expression in leukocytes. Measuring gene expression in single or a collection of pure CTCs (23) could give a more comprehensive and reliable sensitivity profile. Future research should also focus on diagnostic leukapheresis (DLA), since with this technique large amounts of CTCs can be obtained, and on generating organoids from CTCs to test drug sensitivity (24). Large amounts of patient derived and pure materials, in combination with techniques as single CTC genomics and transcriptomics, are promising tools to generate predictive sensitivity profiles.

Despite the failure to meet the primary endpoint, to the best of our knowledge, this is the largest group of MBC patients treated with cDDP monotherapy thus far. A few studies have investigated cDDP monotherapy for breast cancer in the neoadjuvant or metastatic setting, but these were all smaller (3–7, 25–29). In these studies, a variety of RRs have been reported. In patients who received prior treatment for metastatic disease (patients were treated with cDDP in the second to fifth line of therapy), average RRs were 9% (range 0-21%) (5, 6, 27–29), which is comparable with the 7% PR as best observed response in our study.

In our study, 33 (56%) patients had SD as best observed response. The median PFS of all patients was 2.5 months and the median OS 6.9 months, which is as expected in this heavily pretreated group of patients. Cortes and colleagues conducted a study in a patient group that is close to our cohort of patients for comparing outcome to cDDP to other treatments given in this setting (2). They investigated eribulin treatment (*n*=503) versus treatment of choice of the treating physician (TPC) in heavily pretreated patients with locally recurrent or metastatic breast cancer. This TPC (*n*=247) consisted of 25% vinorelbine, 19% gemcitabine, 18% capecitabine, 15% taxanes, 10% anthracyclines and 10% other chemotherapies. In the eribulin group RRs of 12% were found and in the TPC group of 5%. Stable disease was found in 44% of the eribulin group and in 45% of the TPC group. Median PFS for eribulin was 3.7 months and the median PFS in the TPC group was 2.2 months (2). Furthermore, Bardia and colleagues compared sacituzumab govitecan (n=235) (an antibody–drug conjugate composed of an antibody targeting the human trophoblast cell-surface antigen 2) with single-agent chemotherapy of TPC (n=233 received eribulin, vinorelbine, capecitabine or gemcitabine) in relapsed or refractory metastatic breast cancer patients (progression on >2 previous standard chemotherapy regimens, including a taxane) (30). However, this study was performed in patients with a triple-negative breast cancer only. Median PFS was 5.6 months for sacituzumab govitecan and 1.7 months in the TPC group and median OS 12.1 months and 6.7 months, respectively. In total, 35% of the patients that received sacituzumab govitecan had an objective response and 5% in the chemotherapy group. So, comparing this to our data, similar RRs were found for cDDP treatment in heavily pretreated patients compared to the other chemotherapy regimens given.

In the search for markers which predict response to cDDP therapy, impact of the tumor subtypes on outcome was assessed in exploratory analysis. As commonly done, these subtypes were determined on primary tumor tissue. It should be kept in mind that during the course of disease and under treatment pressure the molecular characteristics determining these subtypes can change. To the best of our knowledge, we are the first to measure CTCs in heavily pretreated MBC patients who received cDDP therapy. In accordance with data from MBC patients who were not heavily pretreated (13), CTCs were an independent prognostic marker for both PFS and OS in our set of MBC patients receiving cDDP. While literature shows that patients with TNBC and/or a BRCA1 mutation may have a better response to platinum treatment with RRs up to 80% (25, 26, 31), our data did not show an improved PFS or OS in TNBC patients nor in BRCA-positive patients. However, for the majority (41 out of 65 (63%)) of the patients the BRCA status was unknown, resulting in a very low power to detect an effect. For future research, it would be interesting to investigate in a set of BRCA mutation carriers whether a gene expression profile in CTCs can discriminate patients with a good from those with a poor outcome. And also, it would be interesting to look at homologous recombination deficiency (HRD) since HRD can identify TNBC tumors that are more likely to respond to platinum-containing therapies (32).

As mentioned before, toxicity might be one of the reasons that cDDP is not widely considered as a treatment option in MBC. Treatment with cDDP in this study seemed to be tolerable with 9% (6/65) of the patients discontinuing cDDP treatment due to objectified toxicity and 10% of the patients experiencing grade 3-4 toxicity related to the cDDP treatment.

## Conclusions

In conclusion, the CTC-cDDP-sensitivity profile derived from breast cancer cell lines was unable to select patients responding to cDDP therapy. In an unselected group of heavily pretreated MBC patients, cDDP monotherapy yields outcomes comparable to the outcomes achieved with other regimens which are used in this setting. Furthermore, the prognostic value of CTC enumeration was also found in cDDP-treated MBC patients. Further studies are needed to identify biomarkers which can be used in the clinic to specifically select patients for platinum-compounds.

## Data Availability Statement

The datasets presented in this article are only available from the corresponding author on reasonable request.

## Ethics Statement

The studies involving human participants were reviewed and approved by Medical Research Ethics Committee of the Erasmus MC. The patients/participants provided their written informed consent to participate in this study.

## Author Contributions

Conceptualization: AS, NB, AJ, JK, SS. Data curation: IK, AS, NB, WP-S, LA, CB, MV, AJ, PH, FJ, JK. Formal analysis: IK, AS, LA, EO-H. Funding acquisition: IK, NB, JM, SS. Investigation: IK, NB, LA, JK, JM, SS. Methodology: NB, JK, JM, SS. Project administration: IK, AS, NB. Supervision: JM, SS. Visualization: IK, AS. Writing – original draft: IK, AS. Writing – review: NB, WP-S, LA, CB, MV, EO-H, AJ, PH, FJ, JK, JM, SS. Writing – editing: IK. All authors contributed to the article and approved the submitted version.

## Funding

This work was supported by A Sisters Hope. SS, AS and JM were supported by Cancer Genomics Netherlands (CGC.nl) funded by the Netherlands Organization for Scientific Research (NWO).

## Conflict of Interest

The authors declare that the research was conducted in the absence of any commercial or financial relationships that could be construed as a potential conflict of interest.

## Abbreviations

AE, Adverse event; BR, Bloom-Richardson; cDDP, Cisplatin; CR, Complete Response; CT, Computed Tomography; CTC, Circulating Tumor Cell; CTCAE, National Cancer Institute Common Toxicity Criteria; DLDA, Diagonal Linear Discrimination Analysis; ER, Estrogen Receptor; HBD, Healthy Blood Donor; MBC, Metastatic Breast Cancer; METC, Medical Research Ethics Committee; OS, Overall Survival; PD, Progressive Disease; PFS, Progression-Free Survival; PR, Partial Response; RECIST, Response Evaluation Criteria in Solid Tumors; RR, Response Rate; RT-qPCR, quantitative Reverse Transcription Polymerase Chain Reaction; SAE, Serious Adverse Event; SD, Standard Deviation/Stable Disease; SRB, Sulforhodamine B; STR, Short Tandem Repeat; TNBC, Triple Negative Breast Cancer; TPC, Treating Physician.
