# Supplementary material for: Prospective Evaluation of a Circulating Tumor Cell Sensitivity Profile to Predict Response to Cisplatin Chemotherapy in Metastatic Breast Cancer Patients
Source: Front Oncol. 2021 Jun 25;11:697572. doi: 10.3389/fonc.2021.697572 (PMC8269318; doi:10.3389/fonc.2021.697572)
Supplement: Supplementary file 1 [file DataSheet_1.docx]

**Supplementary Figure 1**. Cisplatin sensitivity of cultured cell line cells

**A)**

**
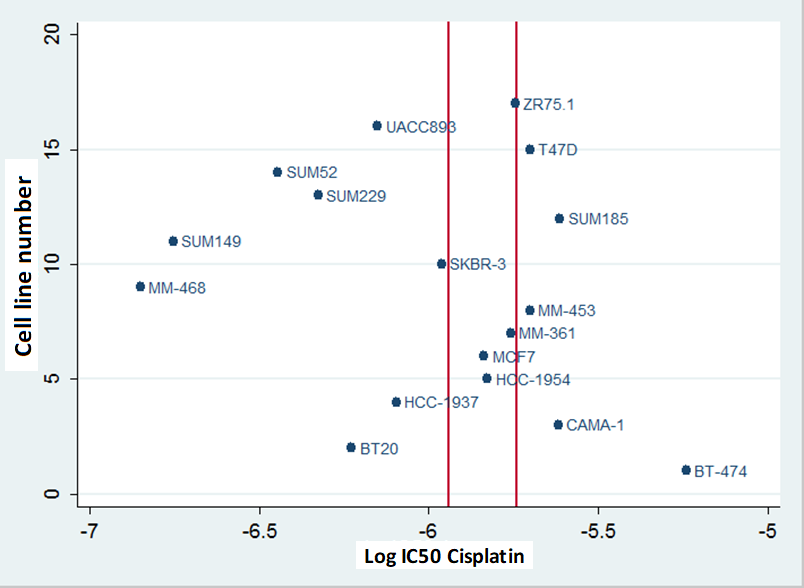
**

**B)**

*Cells were cultured in their respective growth medium in the absence and presence of increasing concentrations of cisplatin.* ***A****) an example of estimating cisplatin sensitivity in BT20 cells.* ***B****) the resulting data of all cell lines summarized. For this, the mean IC50 data of two independent cell culture experiments was used. Cell lines outside the median ± 2SD, depicted by the red lines, were considered to be sensitive [at the left side] or insensitive/resistant {right side). The cell line cells between the lines with inconclusive data were not further explored in our spike-in experiments.*
