# Supplementary material for: Prospective Evaluation of a Circulating Tumor Cell Sensitivity Profile to Predict Response to Cisplatin Chemotherapy in Metastatic Breast Cancer Patients
Source: Front Oncol. 2021 Jun 25;11:697572. doi: 10.3389/fonc.2021.697572 (PMC8269318; doi:10.3389/fonc.2021.697572)
Supplement: Supplementary file 2 [file DataSheet_2.docx]

**Supplementary Figure 2.** Distribution of the cell lines


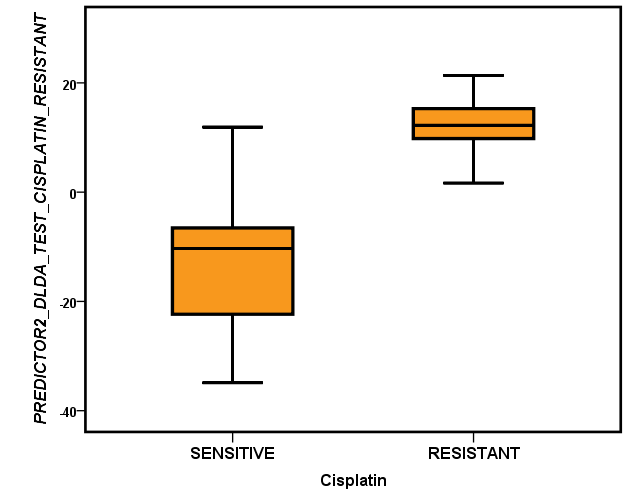


*Distribution of the sensitive and resistant cell lines based on the predictor generated with the DLDA test.*
