# Supplementary material for: Prospective Evaluation of a Circulating Tumor Cell Sensitivity Profile to Predict Response to Cisplatin Chemotherapy in Metastatic Breast Cancer Patients
Source: Front Oncol. 2021 Jun 25;11:697572. doi: 10.3389/fonc.2021.697572 (PMC8269318; doi:10.3389/fonc.2021.697572)
Supplement: Supplementary file 3 [file DataSheet_3.docx]

**Supplementary Figure 3**. PFS and OS in relation to the CTC-sensitivity profile (*n*=58)

**
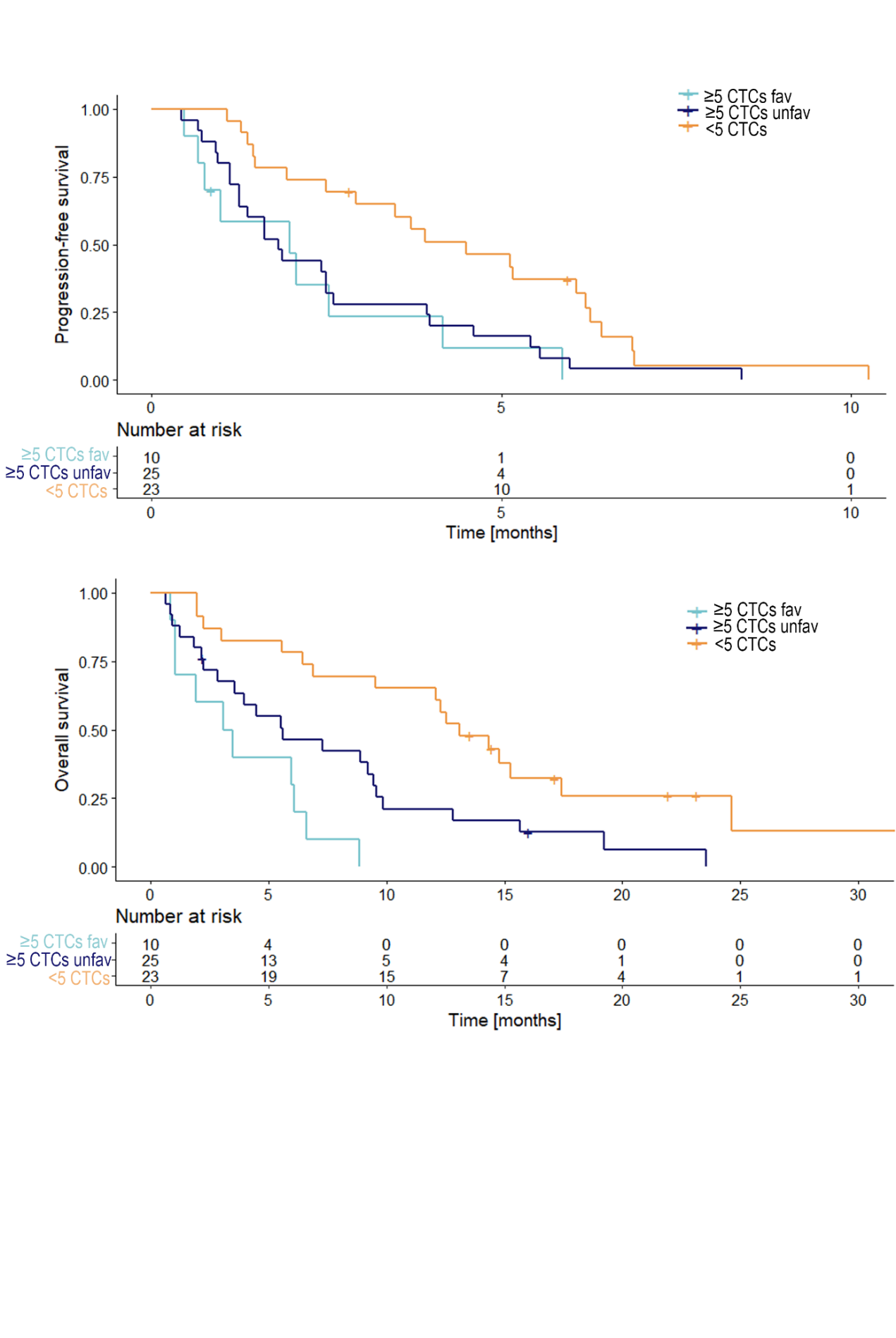
**

**A)**

**B)**

*Kaplan Meier curves of* ***(A)*** *progression-free survival (PFS) and* ***(B)*** *overall survival (OS) in relation to the CTC-cDDP-sensitivity profile. Patients with ≥5 CTCs were divided into the favorable and unfavorable group. Furthermore, patients with <5 CTCs are depicted.*
