# Supplementary material for: Prospective Evaluation of a Circulating Tumor Cell Sensitivity Profile to Predict Response to Cisplatin Chemotherapy in Metastatic Breast Cancer Patients
Source: Front Oncol. 2021 Jun 25;11:697572. doi: 10.3389/fonc.2021.697572 (PMC8269318; doi:10.3389/fonc.2021.697572)
Supplement: Supplementary file 4 [file DataSheet_4.docx]

**
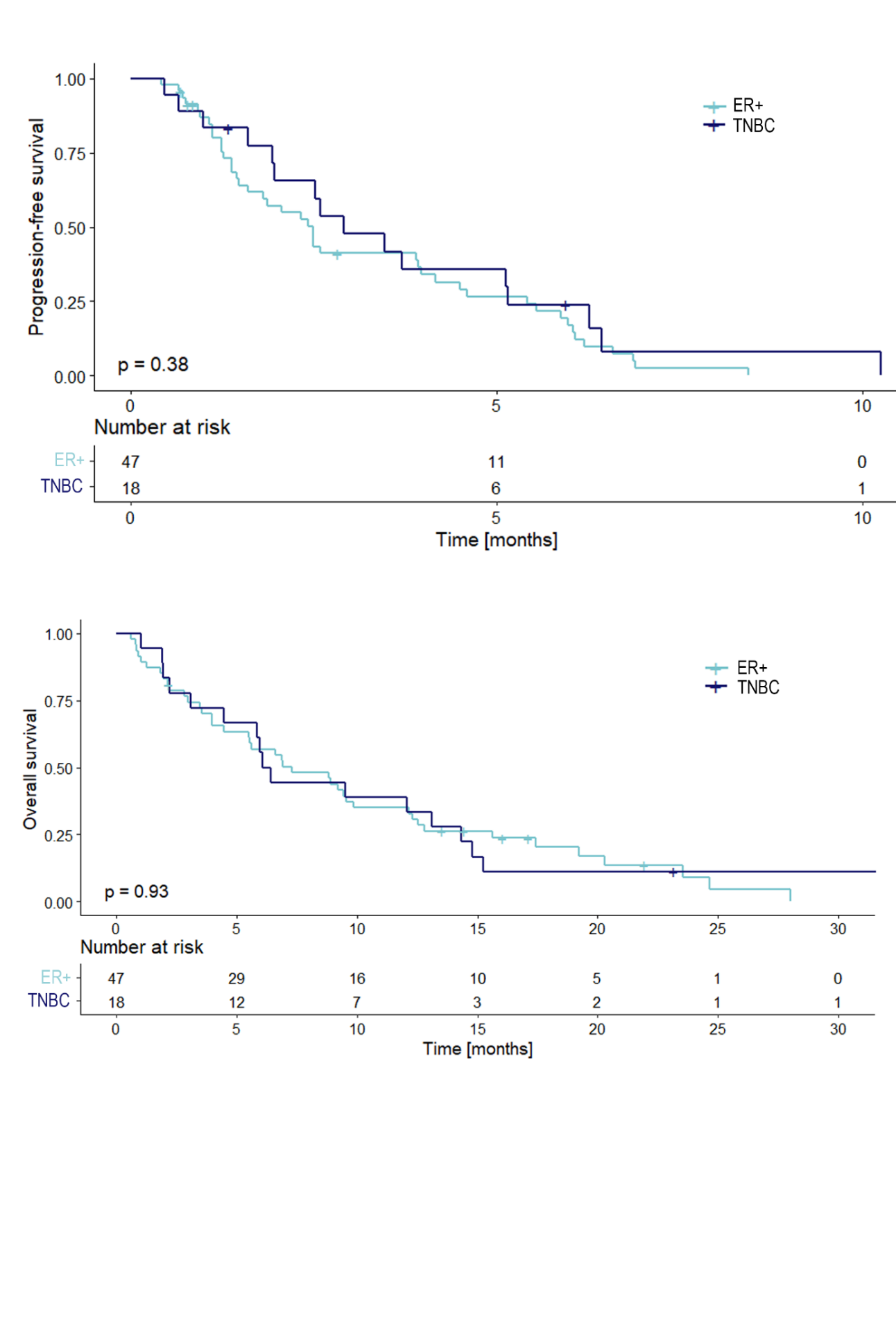
Supplementary Figure 4**. PFS and OS in relation to the breast cancer subtypes (*n*=65)

**A)**

**B)**

*Kaplan Meier curves of* ***(A)*** *progression-free survival (PFS) and* ***(B)*** *overall survival (OS) in relation to the breast cancer subtypes. In light blue the ER-positive patients are depicted, while in dark blue patients with triple negative breast cancer are shown.*
