# Supplementary material for: Prospective Evaluation of a Circulating Tumor Cell Sensitivity Profile to Predict Response to Cisplatin Chemotherapy in Metastatic Breast Cancer Patients
Source: Front Oncol. 2021 Jun 25;11:697572. doi: 10.3389/fonc.2021.697572 (PMC8269318; doi:10.3389/fonc.2021.697572)
Supplement: Supplementary file 5 [file Table_1.docx]

**Supplementary Table 1**. In- and exclusion criteria

| Population |
| --- |
| Female patients with metastatic breast cancer who have been pretreated with at least anthracycline- and taxane-based chemotherapy in the adjuvant and/or metastatic setting |
|  |
| Inclusion criteria |
| Measurable disease according to RECIST 1.1 |
| Age ≥ 18 years |
| WHO performance status ≤2 |
| Adequate haematological functions defined as ANC ≥ 1.0 x 109/L, platelets ≥ 100 x 109/L |
| Adequate renal function defined as creatinine clearance ≥ 60 mL/min (Cockcroft Gault) |
| Patients with reproductive potential must use a reliable method of contraception |
| Written informed consent |
|  |
| Exclusion criteria |
| Other anticancer chemotherapy, use of biological response modifiers, or immunotherapy within two weeks prior to treatment start |
| Hormonal antitumor treatment within one week prior to treatment start |
| Hearing loss of at least Common Terminology Criteria for Adverse Events (CTCAE) grade 2 |
| Neuropathy of at least CTCAE grade 2 |
| Pregnant or lactating patients |
| Serious illness or medical unstable condition prohibiting adequate treatment and follow-up |
| Symptomatic CNS metastases |
| History of psychiatric disorder that would prohibit the understanding and giving of informed consent or adequate follow-up |
