# Supplementary material for: Prospective Evaluation of a Circulating Tumor Cell Sensitivity Profile to Predict Response to Cisplatin Chemotherapy in Metastatic Breast Cancer Patients
Source: Front Oncol. 2021 Jun 25;11:697572. doi: 10.3389/fonc.2021.697572 (PMC8269318; doi:10.3389/fonc.2021.697572)
Supplement: Supplementary file 6 [file Table_2.docx]

**Supplementary Table 2**. Sensitivity and specificity discovery and validation CTC-cDDP-sensitivity profile

| **PREDICTOR_DLDA_TEST CISPLATIN** | **DISCOVERY** | **VALIDATION** |
| --- | --- | --- |
| Sensitivity | 100.00% | 85.71% |
| Specificity | 100.00% | 85.71% |
| Positive predictive value | 100.00% | 85.71% |
| Negative predictive value | 100.00% | 85.71% |
| Disease prevalence | 38.46% | 50.00% |
