# Supplementary material for: Prospective Evaluation of a Circulating Tumor Cell Sensitivity Profile to Predict Response to Cisplatin Chemotherapy in Metastatic Breast Cancer Patients
Source: Front Oncol. 2021 Jun 25;11:697572. doi: 10.3389/fonc.2021.697572 (PMC8269318; doi:10.3389/fonc.2021.697572)
Supplement: Supplementary file 7 [file Table_3.docx]

**Supplementary Table 3**. Line listing of all SAEs

| **Patient** | **Description** | **Cycle** | **Worst grade** | **SAE** | **SUSAR** | **Related** |
| --- | --- | --- | --- | --- | --- | --- |
| P01 | No SAE’s |  |  |  |  |  |
| P02 | Congestive heart failure | 1 | 3 | Yes | No | No |
| P03 | Pneumonia | 2 | 3 | Yes | No | No |
|  | Post obstruction pneumonia | 3 | 3 | Yes | No | No |
| P04 | No SAE’s |  |  |  |  |  |
| P05 | Increased Gamma-GT | 3 | 3 | Yes | No | No |
| P06 | No SAE’s |  |  |  |  |  |
| P07 | No SAE's |  |  |  |  |  |
| P08 | No SAE’s |  |  |  |  |  |
| P09 | Acute kidney failure | 2 | 2 | Yes | No | Yes |
|  | Urinary tract infection | 2 | 2 | Yes | No | No |
| P10 | Anorexia | 2 | 2 | Yes | No | Yes |
|  | Hypoglycemia | 2 | 4 | Yes | No | No |
|  | Nausea | 2 | 2 | Yes | No | Yes |
|  | Pain arm with sensory disorder | 2 | 2 | Yes | No | No |
|  | Weight loss | 2 | 2 | Yes | No | Yes |
| P11 | Muscle weakness right arm | Baseline | 3 | Yes | No | NA |
|  | Nausea | 3 | 3 | Yes | No | No |
|  | Vomiting | 3 | 3 | Yes | No | No |
| P12 | No SAE’s |  |  |  |  |  |
| P13 | No SAE’s |  |  |  |  |  |
| P14 | Anemia | 1 | 3 | Yes | No | No |
|  | Pleuritic pain | 1 | 3 | Yes | No | No |
|  | Nausea | 1 | 2 | Yes | No | No |
|  | Febrile neutropenia | 2 | 3 | Yes | No | No |
|  | Thrombocytopenia | 2 | 4 | Yes | No | No |
|  | Nausea | 2 | 2 | Yes | No | No |
|  | Hepatic failure | 2 | 3 | Yes | No | No |
| P15 | No SAE’s |  |  |  |  |  |
| P16 | Hypokalemia | 1 | 3 | Yes | No | No |
|  | Sepsis | 1 | 4 | Yes | No | No |
|  | Dyspnea | 1 | 3 | Yes | No | No |
|  | Thromboembolic event | 1 | 2 | Yes | No | No |
| P17 | Nausea | 6 | 3 | Yes | No | No |
| P18 | No SAE's |  |  |  |  |  |
| P19 | Diarrhea | 1 | 2 | Yes | No | No |
|  | Nausea | 1 | 2 | Yes | No | No |
| P20 | No SAE's |  |  |  |  |  |
| P21 | Dyspnea | 1 | 3 | Yes | No | No |
|  | Anemia | 1 | 2 | Yes | No | No |
| P22 | Back pain | 1 | 3 | Yes | No | No |
|  | Hypotension | 1 | 2 | Yes | No | No |
|  | Nausea | 1 | 2 | Yes | No | Yes |
|  | Hypotension | 2 | 1 | Yes | No | No |
|  | Nausea | 2 | 1 | Yes | No | Yes |
| P23 | Fatigue | 1 | 3 | Yes | No | No |
|  | Dyspnea | 1 | 3 | Yes | No | No |
| P24 | Abdominal pain | 1 | 2 | Yes | No | No |
|  | Agitation | 1 | 2 | Yes | No | No |
|  | Fecal incontinence | 1 | 1 | Yes | No | No |
|  | Fever | 1 | 1 | Yes | No | No |
|  | Pain thorax | 1 | 2 | Yes | No | No |
|  | Urine incontinence | 1 | 2 | Yes | No | No |
| P25 | No SAE’s |  |  |  |  |  |
| P26 | No SAE’s |  |  |  |  |  |
| P27 | No SAE’s |  |  |  |  |  |
| P28 | No SAE’s |  |  |  |  |  |
| P29 | Dehydration | 4 | 4 | Yes | No | No |
| P30 | Acute kidney failure | 4 | 1 | Yes | No | No |
|  | Fever | 4 | 1 | Yes | No | No |
|  | Hypercalcemia | 4 | 1 | Yes | No | No |
| P31 | Hyponatremia | 1 | 3 | Yes | No | No |
|  | Acute kidney failure | 1 | 1 | Yes | No | No |
| P32 | No SAE’s |  |  |  |  |  |
| P33 | Abdominal pain | 5 | 3 | Yes | No | No |
|  | Hypokalemia | 5 | 3 | Yes | No | Yes |
|  | Hypomagnesemia | 5 | 1 | Yes | No | Yes |
| P34 | Thromboembolic event | 3 | 3 | Yes | No | No |
| P35 | No SAE’s |  |  |  |  |  |
| P36 | Hyperglycaemia | 1 | 3 | Yes | No | No |
|  | Acute kidney failure | 3 | 2 | Yes | No | No |
|  | Acute kidney failure | 4 | 2 | Yes | No | No |
|  | Nausea | 4 | 2 | Yes | No | No |
|  | Vomiting | 4 | 2 | Yes | No | No |
| P37 | Fever | Baseline | 1 | Yes | NA | NA |
|  | Nausea | Baseline | 2 | Yes | NA | NA |
|  | Hypercalcemia | Baseline | 2 | Yes | NA | NA |
|  | Acute kidney failure | Baseline | 1 | Yes | NA | NA |
| P38 | Back pain | 1 | 3 | Yes | No | No |
|  | Radicular pain left leg | 1 | 2 | Yes | No | No |
|  | Nausea | 1 | 2 | Yes | No | No |
| P39 | Acute kidney failure | 2 | 2 | Yes | No | Yes |
|  | Anemia | 2 | 3 | Yes | No | No |
|  | Bleeding lower tr. digestivus | 2 | 4 | Yes | No | No |
|  | Hypocalcemia | 2 | 3 | Yes | No | Yes |
|  | Hypokalemia | 2 | 3 | Yes | No | Yes |
|  | Hypotension | 2 | 4 | Yes | No | No |
|  | Reversible posterior leukoencephalopathy syndrome | 2 | 4 | Yes | No | Yes |
|  | Thrombocytopenia | 2 | 4 | Yes | No | No |
|  | Mucositis | 2 | 3 | Yes | No | Yes |
| P40 | Hypercalcaemia | Baseline | 3 | Yes | NA | NA |
|  | Impending femur fracture | Baseline | 3 | Yes | NA | NA |
|  | Back pain | Baseline | 2 | Yes | NA | NA |
|  | Hypercalcaemia | 1 | 3 | Yes | No | No |
|  | Nausea | 1 | 3 | Yes | No | No |
|  | Hypercalcaemia | 2 | 3 | Yes | No | No |
|  | Nausea | 2 | 3 | Yes | No | No |
| P41 | No SAE’s |  |  |  |  |  |
| P42 | Posterior vitreous detachment | 1 | 1 | Yes | No | No |
| P43 | Anemia | Baseline | 3 | Yes | NA | NA |
|  | Radicular back pain | Baseline | 3 | Yes | NA | NA |
|  | Anemia | 1 | 3 | Yes | No | No |
|  | Dyspnea | 1 | 3 | Yes | No | No |
|  | Pleural effusion | 1 | 3 | Yes | No | No |
| P44 | Congestive heart failure | 1 | 3 | Yes | No | No |
|  | Acute kidney failure | 1 | 1 | Yes | No | Yes |
|  | Hypokalemia | 1 | 1 | Yes | No | No |
| P45 | Cough | Baseline | 2 | Yes | NA | NA |
|  | Hoarseness | Baseline | 2 | Yes | NA | NA |
|  | Pain lymph node metastasis | Baseline | 2 | Yes | NA | NA |
|  | Constipation | 1 | 2 | Yes | No | No |
| P46 | Hyponatremia | 1 | 3 | Yes | No | Yes |
|  | Vomiting | 1 | 2 | Yes | No | Yes |
| P47 | Urinary tract infection | 1 | 3 | Yes | No | No |
|  | Hyponatremia | 1 | 3 | Yes | No | No |
|  | Thrombocytopenia | 1 | 3 | Yes | No | Yes |
|  | Acute kidney failure | 1 | 1 | Yes | No | No |
|  | Dizziness | 2 | 2 | Yes | No | Yes |
| P48 | No SAE’s |  |  |  |  |  |
| P49 | Pleural effusion | Baseline | 3 | Yes | NA | NA |
|  | Palpitations | 1 | 1 | Yes | No | No |
|  | Nausea | 1 | 3 | Yes | No | Yes |
|  | Anorexia | 1 | 3 | Yes | No | Yes |
|  | Fatigue | 1 | 3 | Yes | No | Yes |
| P50 | No SAE’s |  |  |  |  |  |
| P51 | Abdominal pain | 1 | 2 | Yes | No | No |
| P52 | Hearing loss | 4 | 2 | Yes | No | Yes |
| P53 | No SAE’s |  |  |  |  |  |
| P54 | Hematuria | Baseline | 3 | Yes | NA | NA |
|  | Acute kidney failure | 1 | 4 | Yes | No | Yes |
|  | Ascites | 1 | 3 | Yes | No | No |
| P55 | Thromboembolic event | 1 | 2 | Yes | No | No |
| P56 | Nausea | 2 | 3 | Yes | No | Yes |
|  | Pleural effusion | 2 | 2 | Yes | No | No |
|  | Ascites | 2 | 2 | Yes | No | No |
| P57 | No SAE’s |  |  |  |  |  |
| P58 | No SAE’s |  |  |  |  |  |
| P59 | Anemia | Baseline | 2 | Yes | NA | NA |
|  | Leg pain | 2 | 2 | Yes | No | No |
| P60 | No SAE’s |  |  |  |  |  |
| P61 | No SAE’s |  |  |  |  |  |
| P62 | No SAE’s |  |  |  |  |  |
| P63 | No SAE’s |  |  |  |  |  |
| P64 | No SAE’s |  |  |  |  |  |
| P65 | Dyspnea | 1 | 2 | Yes | No | No |
|  | Cough | 1 | 2 | Yes | No | No |

*Line listing of all found SAEs (serious adverse events) in the participating patients. Patient numbers are assigned randomly. Only the red indicated SAEs are related to cDDP therapy. NA = not applicable (baseline SAEs cannot be a SUSAR (suspected unexpected serious adverse reaction) or related with cDDP since these are present before the start of therapy).*
